# Chemometric modeling of larvicidal activity of plant derived compounds against zika virus vector *Aedes aegypti*: application of ETA indices†

**DOI:** 10.1039/c7ra13159c

**Published:** 2018-01-25

**Authors:** Priyanka De, Rahul B. Aher, Kunal Roy

**Affiliations:** Drug Theoretics and Cheminformatics Laboratory, Department of Pharmaceutical Technology, Jadavpur University Kolkata 700 032 India kunalroy_in@yahoo.com kunal.roy@jadavpuruniversity.in https://sites.google.com/site/kunalroyindia/ +91-33-2837-1078 +91 98315 94140

## Abstract

Dengue, zika and chikungunya have severe public health concerns in several countries. Human modification of the natural environment continues to create habitats in which mosquitoes, vectors of a wide variety of human and animal pathogens, thrive, which can bring about an enormous negative impact on public health if not controlled properly. Quantitative structure–activity relationship (QSAR) modeling has been applied in this work with the aim of exploring features contributing to promising larvicidal properties against the vector *Aedes aegypti* (Diptera: Culicidae). A dataset of 61 plant derived compounds reported in previous literature was used in this present study. A genetic algorithm (GA) was used for QSAR model development employing the “Double Cross Validation” (DCV) tool available at http://teqip.jdvu.ac.in/QSAR_Tools/. The DCV tool removes any bias in descriptor selection from a fixed composition of a training set and often provides an optimum solution in terms of predictivity. Simple topological descriptors, the “Extended Topochemical Atom” (ETA) indices developed by the present authors' group, were used for model development. These descriptors do not require pretreatment of molecular structures by conformational analysis or energy minimization before model development, thus saving computational time and resources. They also avoid ambiguities with respect to the existence of compounds in various conformational states leading to the loss of predictive capability in QSAR models. A number of models were generated from GA, and further, the descriptors appearing in the best model obtained from GA were subjected to partial least squares (PLS) regression to obtain the final robust model. The developed model was validated extensively using different validation metrics to check the reliability and predictivity of the model for enhancing confidence in QSAR predictions. Based on the insights obtained from the PLS model, we can conclude that the presence of hydrogen bond acceptor atoms, the presence of multiple bonds as well as sufficient lipophilicity and a limited polar surface area play crucial roles in regulating the activity of the compounds.

## Abstract 2

Dengue, zika and chikungunya have severe public health concerns in several countries. We have developed here a QSAR model for larvicidal activity of plant derived compounds against the vector *Aedes aegypti*.

## Graphical abstract

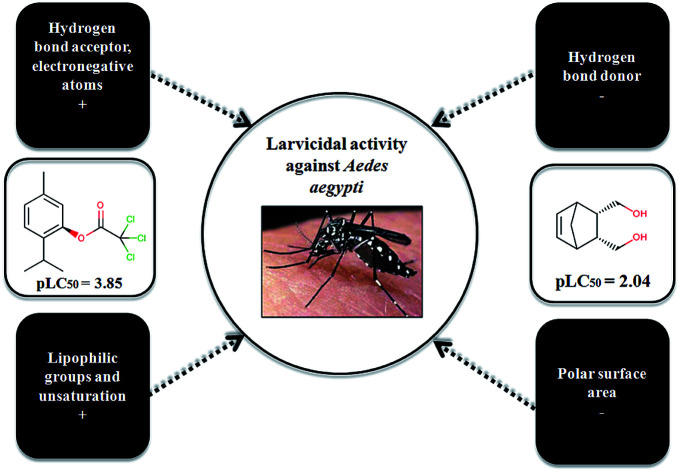

## 1. Introduction

During the past 20 years, there has been a spectacular reappearance or emergence of epidemic arboviral diseases transmitted by mosquitoes affecting both human and domestic animal health.^1^ Human modification of the natural environment continues to create habitats in which mosquitoes, vectors of a wide variety of human and animal pathogens, thrive, which can bring about an enormous negative impact on public health if not controlled properly. Morbidity and mortality have been reported to be increasing at an alarming rate^2^ while a large number of lives are under threat due to mosquito borne diseases, like zika, malaria, chikungunya, dengue and yellow fever. The outbreak of these diseases has been observed mostly in countries like Brazil, Colombia, Mexico, Argentina and India.

Recent reports of local transmission of chikungunya have been made in south-eastern France, with 13 cases (four confirmed, one probable and eight suspected) of people aged between 3 and 77 years.^3^ Also 183 cases have been notified in the Lazio Region of Italy, with 109 confirmed and 74 additional cases.^4^ During the last few years an outbreak of the zika virus transmitted by mosquitoes has been observed in West Africa and in America (in Brazil and Colombia) due to weak health infrastructures and the decline in programmes for mosquito control.^5^ The species responsible for these transmissions were found to be *Aedes aegypti*, *Aedes leucocaelenus*, *Aedes albopictus* and *Aedes sabethes* which proliferate rapidly due to continuous change in the environment leading to invasion of new territories.

The use of safe and efficacious insecticides against the adult and larval populations of mosquito vectors can be an effective way to control the transmission of zika virus and other viruses transmitted by *Aedes* mosquitoes, such as chikungunya and dengue. Pesticides play an effective role in the development of public health by working as a sustainable form of mosquito management.^6^ Synthetic insect repellents like dichloro-diphenyl-trichloroethane (DDT) and *N*,*N*-diethylmetatoluamide (DEET) are used.^6,7^ However, over the time, the vector mosquito has become highly resistant to DDT, which also creates a nuisance by becoming highly accumulated in the environment and producing toxic effects to humans, birds, fish and other animals.^7^

Over the last 50 years, the use of synthetic repellents has been one method of personal protection against mosquito bites. For example, compounds such as dimethyl phthalate (DMP), ethyl hexanediol (EHD) and diethylmetatoluamide (DEET) have been developed for this purpose. DEET, which is still being used worldwide, has some problems with efficacy, irritating effects on the skin, low retention and anaphylactic reactions.^8^

Botanical compounds known as essential oils (EOs) can be an alternative method to conventional pesticides where the former act as repellents, ovicides, adulticides, feeding inhibitors, or attractants for various insect species.^9,10^ A large number of secondary metabolites like alkaloids, terpenoids and phenylpropanoids are found in considerable amounts in various parts of a plant. Compounds like menthol, citronellal, pulegone, linalool and other terpenes have shown insecticidal, fungicidal and larvicidal activities.^11,12^ In a study, oils of 41 plants were evaluated for their effects against *Aedes*, *Anopheles*, and *Culex* larvae, among which 13 oils were found to induce 100% mortality after 24 hours or less in *Aedes aegypti*.^13^

The general aims of developing an ideal repellent are: it should be potent enough to repel a diverse class of vectors, should be effective for about eight to twelve hours, should not cause toxicity to the host, should be non-irritant to the skin and should not bring about systemic toxicity. However, no such compounds could be found with all these properties, and moreover the exploration of new insecticides needs time, a budget and several analytical set-ups.^7^

Quantitative structure–activity relationship (QSAR) modeling is an approach for determining the chemical features contributing to a target activity. This approach can be used for the compounds from plant essential oils with larvicidal activities in order to find a congener with optimum activity.^14^ In the current study, we have utilized a dataset of 61 natural or semi-synthetic compounds with larvicidal activity for QSAR model development, using simple Extended Topochemical Atom (ETA) descriptors developed by the present authors' group.^15,16^ The developed models are aimed at providing statistically robust predictions for the larvicidal activity of the compounds, expressed as the median lethal concentration (LC_50_).

## 2. Materials and methods

### 2.1. The dataset

The experimental larvicidal lethal concentration (LC_50_) values for 61 plant derived compounds were collected from the literature.^17–20^ The concentrations of the chemical in air that kills 50% of the test population during the observation period is the LC_50_ value. The lethal concentration is usually applied for chemicals that are breathed into the body. In all the above-mentioned pieces of research, third instar larvae were used to determine the LC_50_ values of the compounds. The LC_50_ values were converted into their logarithmic scale equivalents (pLC_50_) for the purpose of modeling. The structures of 61 compounds were drawn in the MarvinSketch (version 14.10.27)^21^ application with proper aromatisation and explicit hydrogen addition. In Table S1 in ESI,† various classes of heterogeneous molecular structures involving terpenes, phenylpropanoids, ketones and oxygenated compounds along with their LC_50_ values are given.

### 2.2. Molecular descriptors

In the present work, there is only a single class of descriptors (Extended Topochemical Atom or ETA indices).^22^ The descriptors were calculated using the PaDel-Descriptor software tool.^23^ Variables with constant or near constant values (standard deviation less than 0.0001), descriptors with at least one missing value, descriptors with all values missing and descriptors with (absolute) pair correlation larger than or equal to 0.95 were excluded from the initial pool of descriptors. In the end, a set of 42 ETA descriptors were obtained which were used for model development. Since we have used only 2D descriptors in the present research, the model development does not require any conformational analysis or energy minimization of molecular structures. In addition to 2D descriptors not requiring molecular structure optimization, this approach involves some additional advantages; for instance, topological descriptors are simpler to interpret than geometrical descriptors. In fact, 2D descriptors avoid ambiguities with respect to the existence of compounds in various conformational states, which can lead to the loss of predictive capability in QSAR models.

### 2.3. Dataset division

The whole dataset was divided into training (66% of the all available data points) and test (34%) sets based on a simple and fast algorithm for *k*-Medoids clustering. For this, we employed a software tool “Modified *k*-Medoids” (version 1.2) developed in our laboratory.^24^ The process categorizes a set of objects into clusters, so that the objects within a cluster are similar to each other but are dissimilar to objects present in other clusters.^25^ The indicative objects within a cluster are called medoids. After arranging the whole dataset according to the cluster number with the corresponding activity values, we selected approximately 34% of compounds from each cluster as test set compounds (*n*_test_ = 20) and the remaining 66% as a training set (*n*_train_ = 41). The training set was used for model development and the test set was applied for the purpose of model validation.

### 2.4. Model development

In this study, we have developed a QSAR model using LC_50_ values of the plant derived compounds as the response variable for model development. Initially various statistical tools, such as multiple linear regression (MLR), stepwise regression^26^ and double cross-validation (DCV),^27,28^ were applied to develop the models; finally the most statistically significant and robust model was obtained by a genetic algorithm (GA)^29^ within the DCV tool, followed by partial least squares (PLS) regression analysis.

Double cross-validation^27,28^ is a statistical technique used for the generation and selection of models to produce a better predictive model. The fixed composition of a training set can often influence the selection of descriptors and can lead to a bias in descriptor selection. A double cross-validation method, in which the training set is further divided into ‘*n*’ calibration and validation sets, can result in diverse compositions of the modeling set, thus removing any bias in descriptor selection. In addition, a model with the lowest prediction errors in the validation set is chosen; thus, this procedure is expected to provide an optimum solution in terms of predictivity in most cases. The tool comprises two nested cross-validation loops recognized as internal cross-validation and external cross-validation loops. In the external loop, the compounds in the dataset are divided into training set compounds and test set compounds. The training set compounds are involved in the internal loop for the purpose of model development and model selection, and the test set is used solely for the intention of checking model predictivity. In the internal loop, the training set is further repetitively split into calibration and validation sets by employing the *k*-fold cross-validation technique (in this study, *k* = 10)^27^ and producing *k* iterations to construct calibration and validation sets. In the end, the best models are selected based on various validation metrics.

The double cross-validation technique in MLR model building and selection is a better choice compared to the conventional hold-out method. In the hold-out method, the composition of the training set remains the same, so there is a chance of bias in the descriptor selection. On the other hand, in the DCV method, the training set is further divided into ‘*n*’ calibration and validation sets resulting in diverse compositions. So, there are more chances for optimal selection of descriptors for model development.

PLS regression is a generalization of multiple linear regression (MLR).^30^ PLS provides an approach to the quantitative modeling of the often complex relationships between predictors, *X*, and responses, *Y*, and it is more general and robust than MLR. We performed PLS regression for development of the final model. We used the set of 5 descriptors from the previous step (GA-MLR) and ran PLS, which can handle overfitting and extensive noise during predictive model development. Information about the original variables is stored in latent variables (LV) generated by PLS. Although we used 5 descriptors in our model, it should be noted that PLS modeling is more robust than multiple linear regression, and it uses a reduced number of regression variables (latent variables, which are functions of the original variables). In our case, we used only 3 latent variables. This means that the actual number of regression variables is only 3 (and not 5) allowing an acceptable number of degrees of freedom.

### 2.5. Statistical validation metrics

In this present study, we employed multiple approaches for the evaluation of model quality, for measurement of the fitness, stability, robustness and predictivity of the developed model. The determination coefficient (*R*^2^)^28^ is a measure of goodness-of-fit whereas internal validation (which deals with the predictive ability of the model based on training set compounds) is usually determined by a cross-validated correlation coefficient, *Q*_LOO_^2^ (leave-one-out). *Q*^2^ provides a measure of model robustness, but is not sufficient to determine the performance of the model when new sets of compounds are employed. The external validation of the model was estimated using various parameters, *Q*_*F*_1__^2^ and *Q*_*F*_2__^2^.^31^ The external validation deals with the predictive ability of the model for the test set compounds. Additionally, the root mean square error (RMSE)^32^ was estimated, which summarizes the overall error. We also included the values of standard error of estimate (*s*) and variance ratio (*F*) at the specified degrees of freedom (df) for the training set, to indicate the quality of fit and robustness of the regression coefficients of the developed model, respectively.

## 3. Results and discussion

In the current study, we have developed a PLS regression model using descriptors selected in GA-MLR employed in the DCV tool, as described in the Materials and methods section. The statistical quality of the model developed was sound. The final PLS model developed with five descriptors using three LVs is depicted below:1
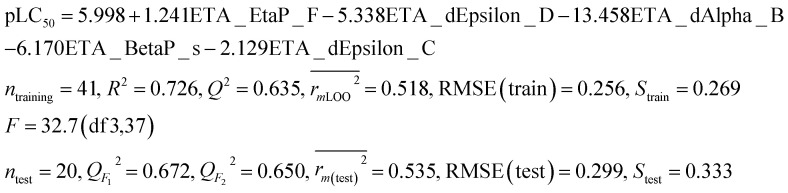


The model showed acceptable values of the coefficient of determination *R*^2^ (0.726) and cross-validated correlation coefficient (LOO–*Q*^2^ = 0.635), and a low standard error of estimate (*S*), signifying the statistical reliability of the model. The significant *F* value (at *p* < 0.05) suggests the robustness of the regression coefficients. The predictivity of the model was judged by means of predictive *R*^2^ (*R*_pred_^2^) or *Q*^2^*F*_1_ (*Q*^2^*F*_1_ = 0.672), which shows a moderate predictive ability for the model. The values of the descriptors appearing in eqn (1) for both training and test set compounds along with model derived (computed) response values are provided in Table S2 in ESI.†

**Fig. 1:**
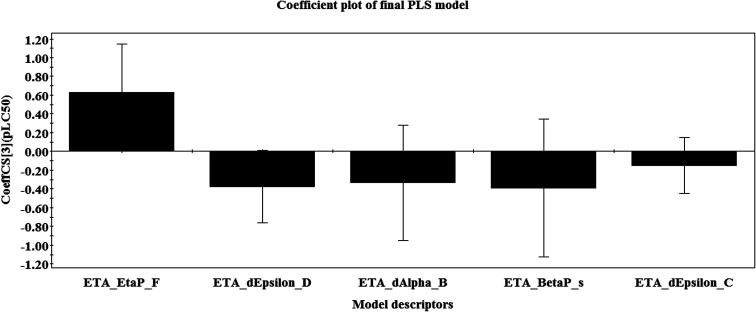
Regression coefficient plot of the final PLS model.

The regression coefficient plot^30^ (Fig. 1) gives knowledge about the positive or negative contribution of descriptors towards the activity of the compounds. A descriptor with a positive correlation coefficient (*i.e.*, ETA_EtaP_F) signifies that as the descriptor value increases, the larvicidal activity value also increases, whereas a descriptor with a negative coefficient (*i.e.*, ETA_dEpsilon_D, ETA_dAlpha_B, ETA_BetaP_s, ETA_dEpsilon_C) indicates that as its value increases, the larvicidal activity decreases.

**Fig. 2:**
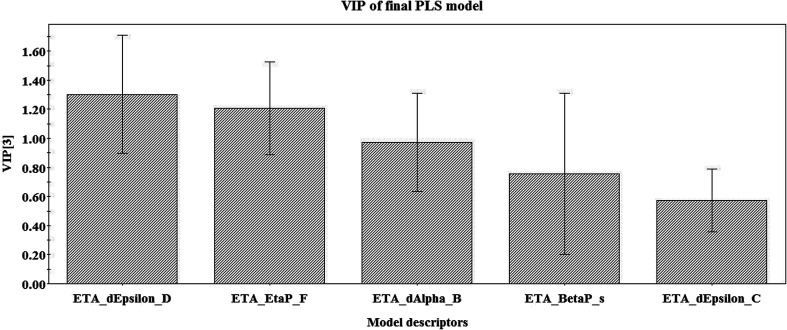
Variable importance plot of the final PLS model.

From the variable importance plot (VIP) (Fig. 2), the significance of each of the descriptors obtained in the final PLS model can be described for their importance to the larvicidal activity of the compounds. The most and the least important descriptors contributing to the larvicidal activity of the used compounds can be identified with the help of this plot (Fig. 2). A variable with VIP score >1 shows higher statistical significance as compared with one with a low VIP value.^33^ The descriptors are arranged in the plot according to their importance (maximum contribution to minimum contribution) and their significance level is found to be in the following order: ETA_dEpsilon_D, ETA_EtaP_F, ETA_dAlpha_B, ETA_BetaP_s and ETA_dEpsilon_C.

**Fig. 3:**
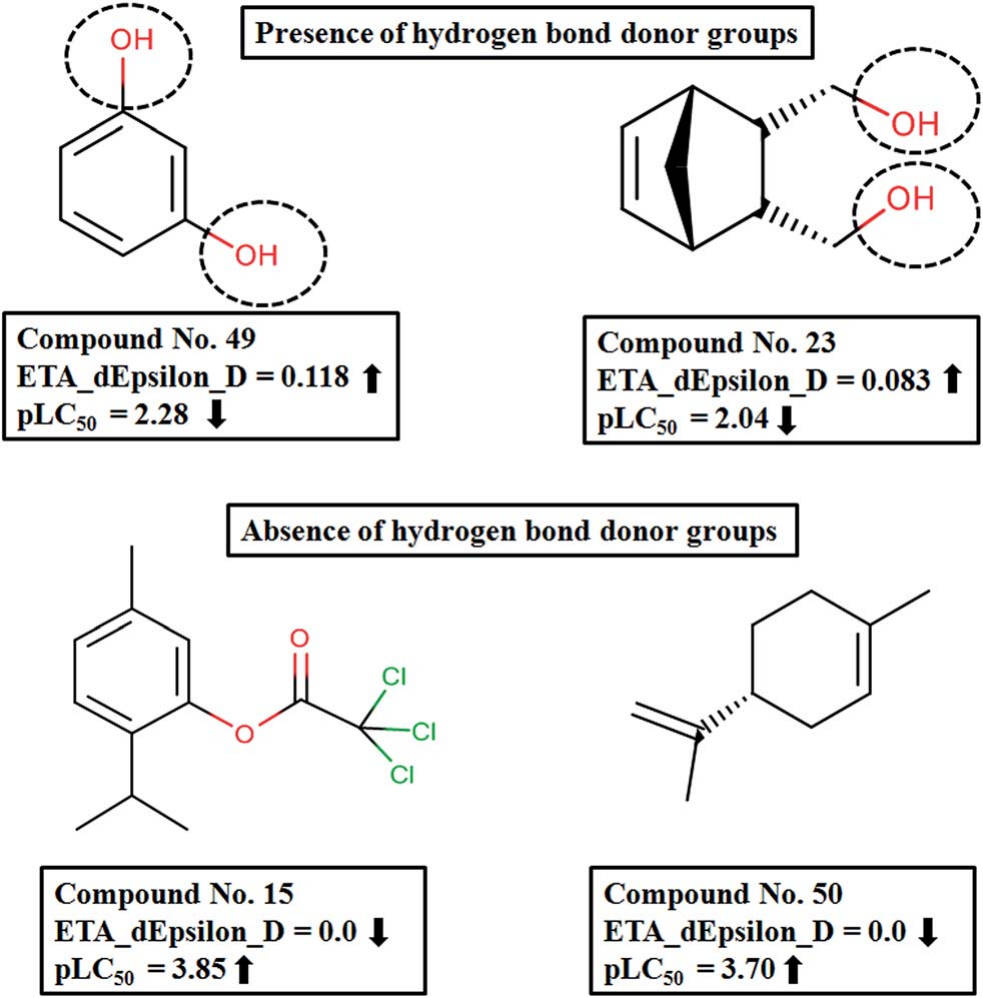
Contribution of ETA_dEpsilon_D to pLC_50_ of the compounds.

The descriptor contributing most to the response is ETA_dEpsilon_D, which is a measure of contribution of hydrogen bond donor atoms, *i.e.*, the presence of groups such as –OH, –NH_2_, –SH *etc.* The negative coefficient of the descriptor shows that there will be a decrease in the desired activity of the compound with an increase in descriptor value, *i.e.*, an increase in the number of hydrogen bond donor atoms. Compounds like 49 (resorcinol), 23 (5-norbornene-2-*endo*-3-*endo*-dimethanol) and 35 (4-hydroxy-3-methoxy-benzenepropanol) have a higher number of hydrogen bond donor atoms contributing to lower activity values, whereas compounds like 15 (thymyl trichloroacetate), 50 (*R*-limonene) and 10 (carvacryl benzoate) have low ETA_dEpsilon_D values leading to higher activity. The effect of the ETA_dEpsilon_D descriptor on the activity of the compounds is depicted in Fig. 3.

**Fig. 4:**
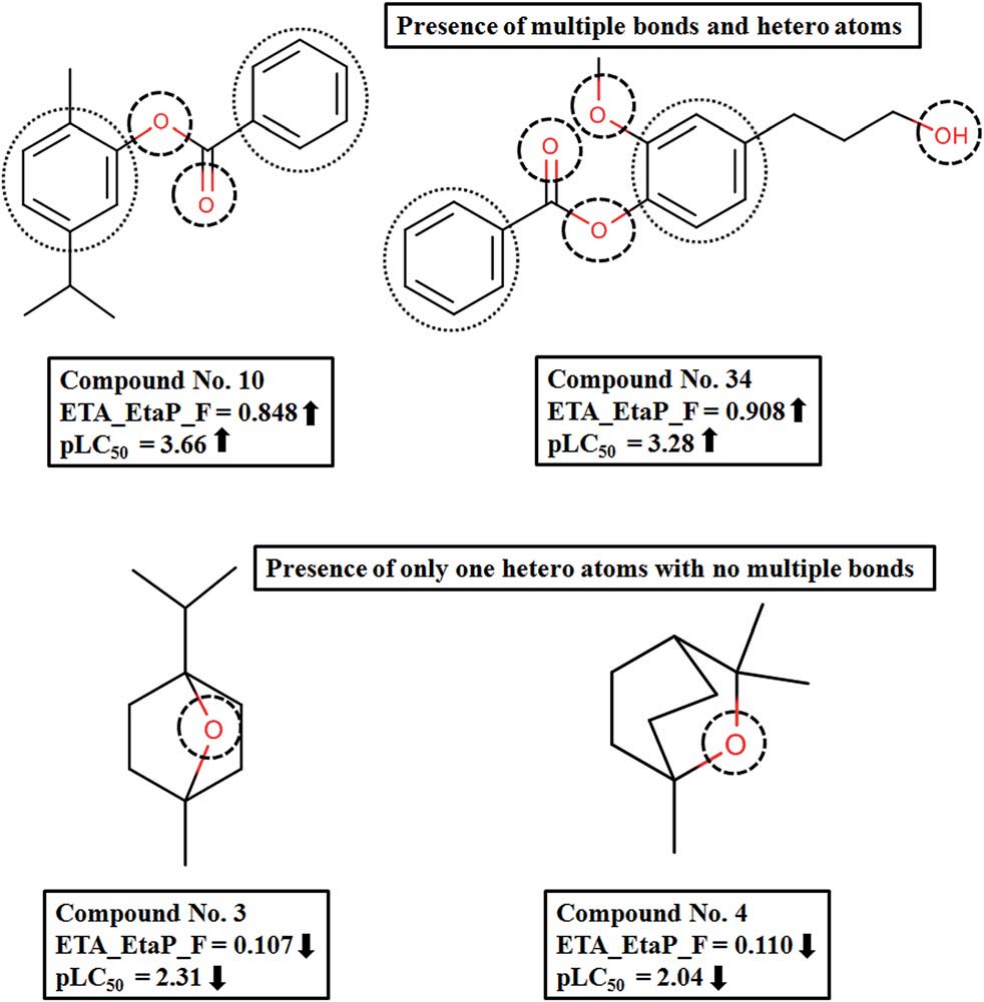
Effect of ETA_EtaP_F on pLC_50_ of the compounds.

The next most important descriptor is ETA_EtaP_F, which is a functionality index relative to molecular size. It gives a measure of the presence of heteroatoms and multiple bonds. The positive regression coefficient of the descriptor denotes that an increased number of heteroatoms and multiple bonds will increase the larvicidal activity against the *Aedes* mosquito. In compounds like 10 (carvacryl benzoate), 17 (thymyl benzoate) and 34 (1-benzoate-2-methoxy-4-(3-hydroxypropyl)-phenol), the number of heteroatoms (like oxygen) and multiple bonds (as in benzene rings) are higher; accordingly the descriptor values are also higher, contributing to increased activity. On the other hand, compounds like 3(1,4-cineole) and 4(1,8-cineole) have low descriptor values, thus leading to lower activity (Fig. 4). From these observations, we can conclude that hydrophobicity is important for larvicidal activity.

**Fig. 5:**
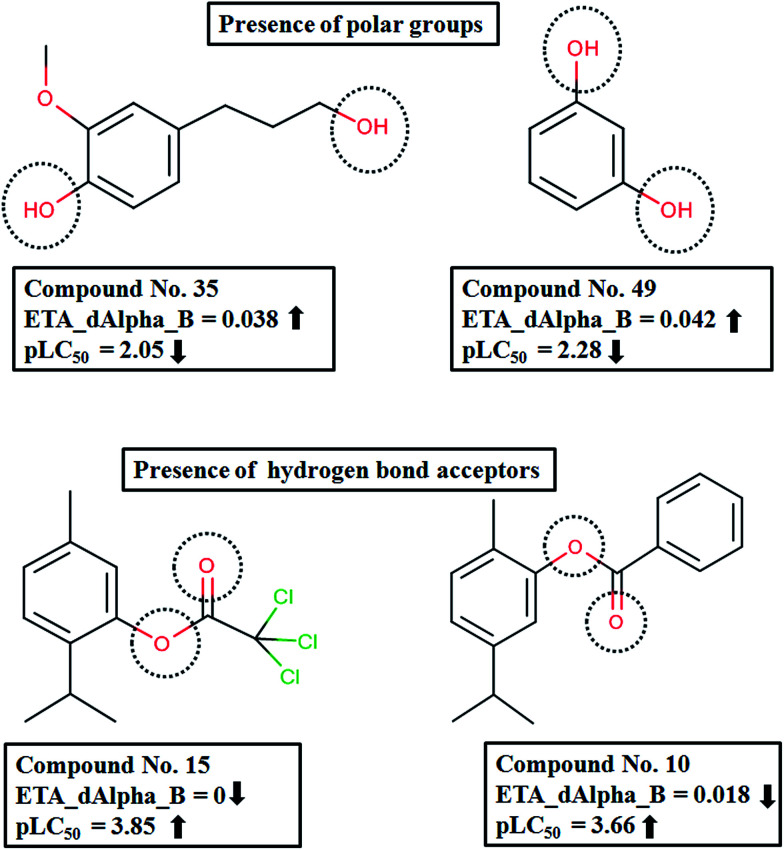
Contribution of ETA_dAlpha_B to pLC_50_ of the compounds.

The descriptor ETA_dAlpha_B(Δα_↓_B) is the next most important descriptor, which is a measure of polar surface area. The negative contribution of this descriptor indicates that the presence of polar groups is detrimental to the activity, as shown in compounds like 35 (4-hydroxy-3-methoxy-benzenepropanol) and 49 (resorcinol). In contrast, compounds like 15 (thymyl trichloroacetate), 10 (carvacryl benzoate) and 14 (thymyl chloroacetate) which have hydrogen bond acceptor atoms have higher activity (Fig. 5).

The fourth important descriptor is ETA_BetaP_s (Σ*β*), which is the sum of the *β* values for all the sigma bonds (VEM sigma contribution)^15,34^ relative to the number of vertices.Σ*β*′_s_ = Σ*β*_s/_*N*_v_

**Fig. 6:**
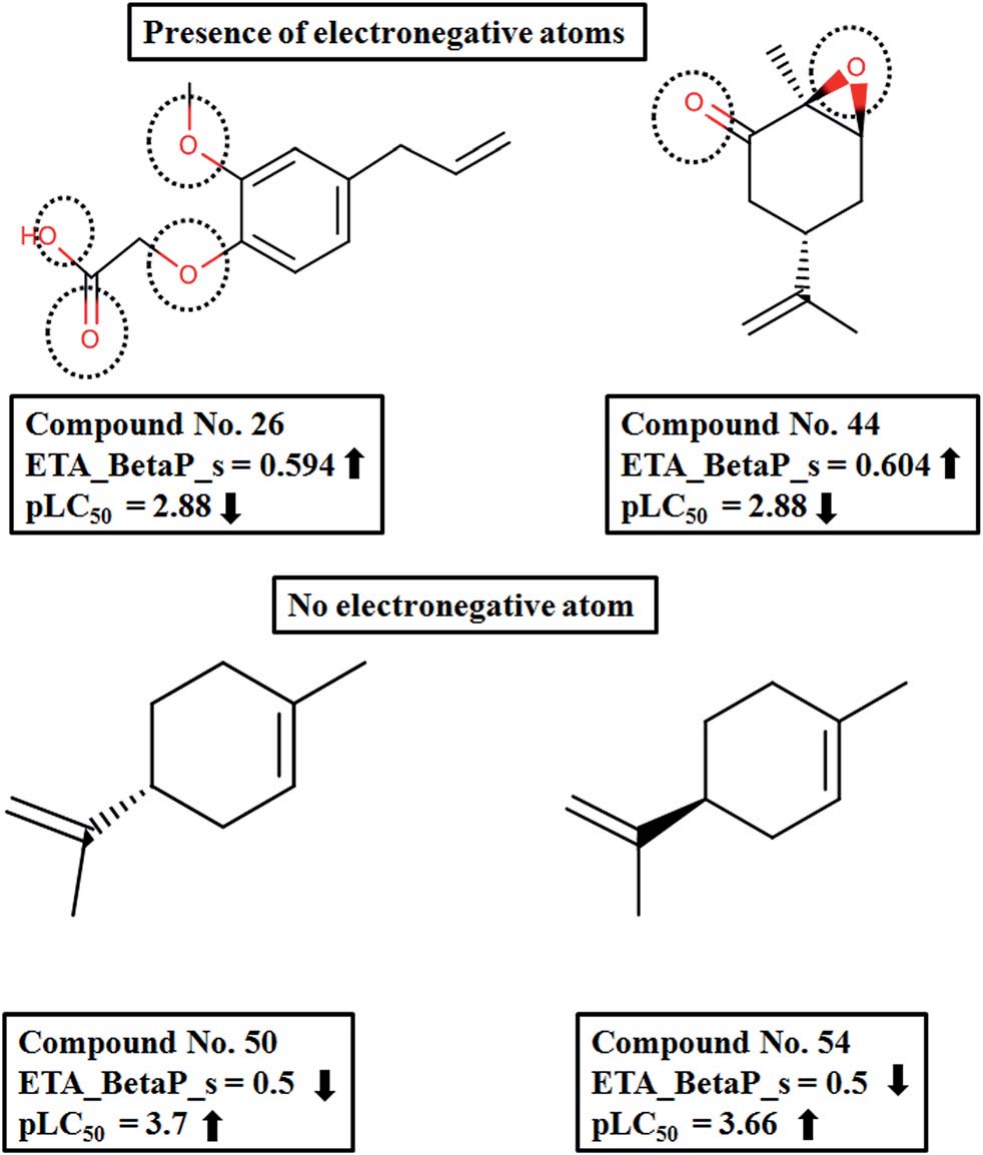
Contribution of ETA_BetaP_s on pLC_50_ of the compounds.

The descriptor ETA_BetaP_s gives a measure of the electronegative atom count of the molecule relative to the molecular size. The negative contribution suggests that with an increase in the descriptor value the activity will decrease. From the above equation, the descriptor values obtained can be justified.^35^ According to the ETA scheme, the sigma contribution of two bonded atoms with similar electronegativity is 0.5 and that for ions with different electronegativity is 0.75. Therefore, considering the relative values (relative to the number of vertices), we can see that in compounds with a higher number of heteroatoms like 26 (2-[2-methoxy-4-(2-propen-1-yl)phenoxy] acetic acid) and 44 (1,2-carvone oxide), the descriptor values are higher (higher sigma contribution). Also the contribution of the descriptor to the activity is also well explained by these compounds, since their activity values are low. Next, if we consider compounds like 50 (*R*-limonene) and 54 (*S*-limonene), which have a nonfunctional carbocyclic skeleton, they have lower descriptor values and consequently their activity values are higher (Fig. 6).

The descriptor with the least importance is ETA_dEpsilon_C(Δ*ε*_C_), which is a measure of electronegativity. The descriptor can be expressed as Δ*ε*_C_ = *ε*_3_ − *ε*_4_, where the terms *ε*_3_ and *ε*_4_ can be defined as:

(i) *ε*_3_: sum of epsilon (*ε*) values relative to the total number of atoms (*N*_R_) including hydrogens in the connected molecular graph of the reference alkane. A reference alkane of a molecule corresponds to a structure where all heteroatoms are replaced with carbon atoms and multiple bonds (covalent) with single bonds.^34^
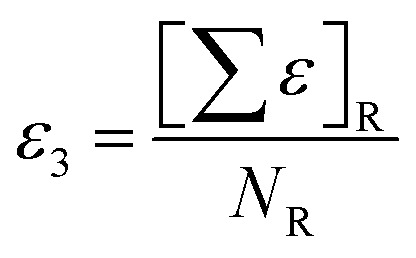
(ii) *ε*_4_: Sum of epsilon (*ε*) values relative to the total number of atoms (*N*_ss_) including hydrogen for a saturated carbon skeleton moiety of the normal molecule, *i.e.*, with carbon–carbon multiple bonds considered as single bonds.^35^
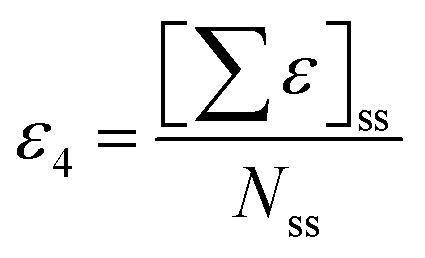


**Fig. 7:**
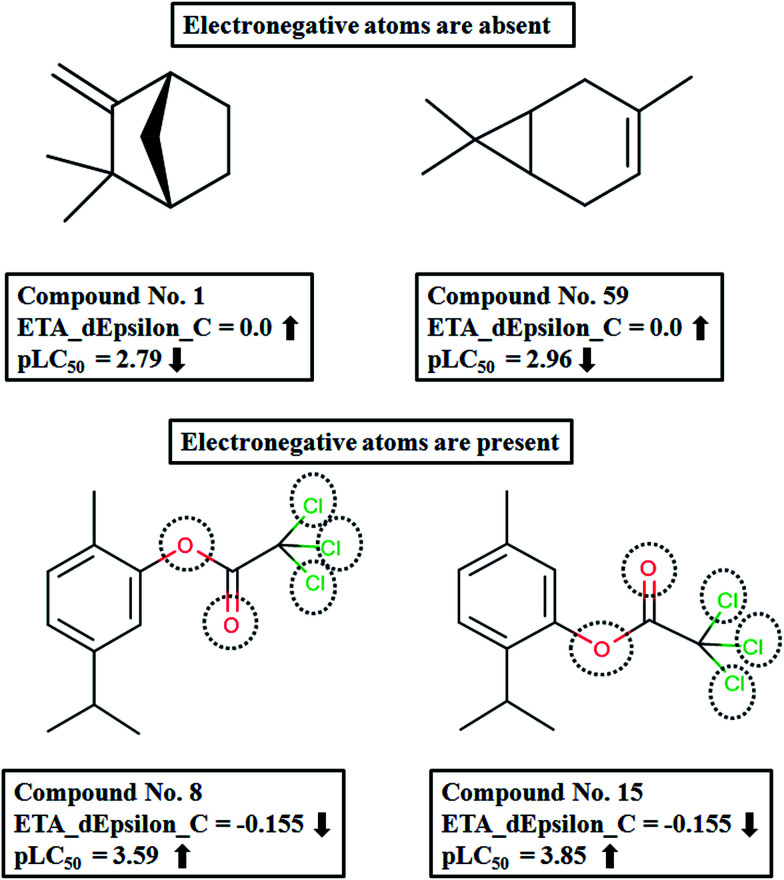
Effect of ETA_dEpsilon_C on pLC_50_ of the compounds.

This descriptor shows a negative influence on the pLC_50_ values; thus an increase in the ETA_dEpsilon_C value will result in a decrease in the response and *vice versa*. In compounds 1 ((−)-Camphene) and 59 (3-Carene), there is an absence of any electronegative atoms and the reference alkane and the saturated carbon skeleton for these two compounds will be the same. Therefore, the values for *ε*_3_ and *ε*_4_ will be the same and hence their difference, Δ*ε*_C_, is zero for both compounds. On the other hand, compounds like 8 (carvacryl trichloroacetate) and 15 (thymyl trichloroacetate), which possess a considerable number of electronegative atoms (five electronegative atoms in both cases), will have higher *ε*_4_ values than *ε*_3_ making Δ*ε*_C_ negative (Fig. 7).

### 3.1. Score plot of the PLS model

**Fig. 8:**
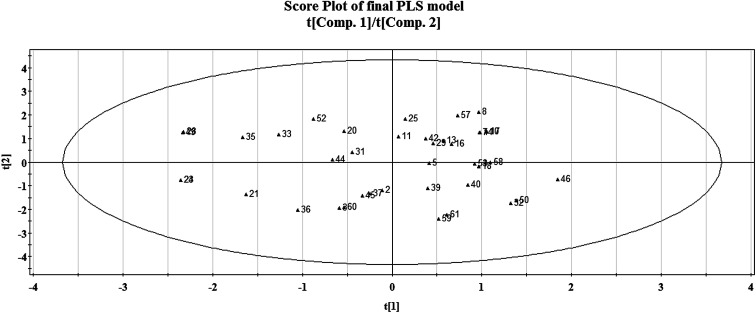
Score plot of the final PLS model.

The distribution of the compounds in the latent variable space as defined by the scores is expressed in a score plot, as given in Fig. 8. Here, we have plotted the scores of the first two components t1 and t2. The ellipse indicates the applicability domain of the model, as defined by Hotelling's t2. Hotelling's t2 is a multivariate generalization of Student's *t*-test. It provides a check for compounds adhering to multivariate normality.^36^ In this plot, compounds which are situated near each other have similar characteristics or properties, whereas compounds which are far from each other have dissimilar properties with respect to their larvicidal activity against the zika vector. For example, compounds which are located in the upper right hand corner like 42 (1,2-dimethoxy-4-(2-propen-1-yl)-benzene) and 29 (1-ethoxy-2-methoxy-4-(2-propen-1-yl)-benzene) have some similarity in properties whereas compounds which are far from each other like those in the lower left hand corner (for example compound number 21 or 5-norbornene-2-ol) and upper right hand corner (for example compound number 8 or carvacryl trichloroacetate) represent heterogeneity in the property space. The compounds which are close to the centre of the plane have average properties. Since there are no compounds present outside the ellipse, we can conclude that there are no outliers according to this method.

### 3.2. Loading plot of the PLS model

**Fig. 9:**
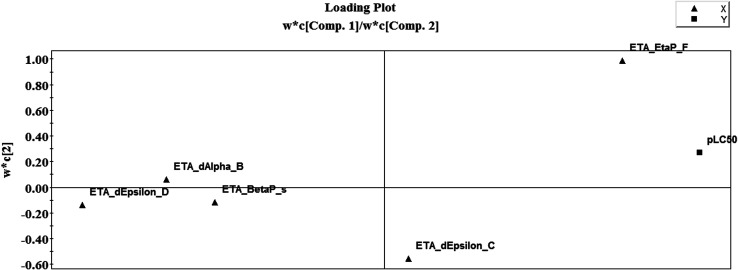
Loading plot of the final PLS model.

A loading plot of a PLS model (Fig. 9) gives the relationship between *X*-variables and *Y*-variables, as shown in Fig. 9, where five *X*-variables and one *Y*-variable (pLC_50_) are shown. The loading plot was developed using the first two components. The loading plot gives us an insight into how the different variables produce an impact on the model and which variable produces the maximum footprint. For interpretation of the PLS model, we should consider the distance from the plot origin. Similar types of variables contributing similar information are grouped together and are correlated. The variables which are situated far away from the plot origin are considered to have a stronger impact on the model for that particular variable. The algebraic sign of the PLS loading is also taken into account, which gives important information about correlation among the variables. The *X*-variable ETA_EtaP_F is influential for the *Y*-variable pLC_50_ because of its closeness to the *Y*-variable. Hence, if the numerical value of this descriptor increases, the larvicidal activity against the *Aedes* mosquito will also increase. In the case of the descriptor ETA_dEpsilon_D, which is present on the opposite side of the plot origin with respect to pLC_50_, this suggests that an increase in ETA_dEpsilon_D value will result in a decrease in activity. From the loading plot, we can also identify the weighting of the *X*-variables based on the first component and second component. From weighting (Table 1) analysis, we can conclude that component 1 considers the hydrogen bonding property of compounds and component 2 considers the electron richness of the compounds.

**Table 1:** Weightage of descriptors for first two PLS components

Descriptors	Weightage based on the first two components	
	Component 1	Component 2
ETA_EtaP_F	0.102729	0.709627
ETA_dEpsilon_D	−0.765005	0.100524
ETA_dAlpha_B	−0.644193	0.384778
ETA_BetaP_s	−0.405975	0.241983
ETA_dEpsilon_C	0.385227	−0.568717

### 3.3. Applicability domain of PLS model

The applicability domain (AD) gives a theoretical region in chemical space defined by the respective model descriptors and responses in which the predictions are reliable.^37^ The AD assessment of the proposed model for PCPs was performed according to the DModX (distance to model) in the *X*-space approach using SIMCA-P^38^ software. From Fig. S1 (in ESI†) we can see that there is only one outlier to be found in the training set, *i.e.*, compound 8 (or carvacryl trichloroacetate) and one compound outside the AD, *i.e.*, compound 15 (or thymyl trichloroacetate) at a 99% confidence level (D-critical = 0.00999898).

### 3.4. Randomization model of PLS model

The statistical significance of the model is analyzed by randomization plot (Fig. S2 in ESI†). The randomization plot has been developed in order to confirm that the model is not the result of any chance correlation.^39^ In randomization, a number of models are generated by permuting different combinations of *X* or *Y* variables based on the fit of the reordered model. In our study, for the training set, the *X* data remained intact and the *Y* data were shuffled randomly (*Y*-randomization), and the model was fitted to the permuted data and compared with the best fit. The number of permutations can vary; here we used 100 permutations. The basic statistics of randomization models (*Q*^2^ and *R*^2^) should be poor and not within the range of those for acceptable regression models. Otherwise, each resulting model may be considered as a chance correlation.^40^ The value of the *R*_*Y*_^2^ intercept should not exceed 0.3 and the value of the *Q*_*Y*_^2^ intercept should not exceed 0.05. The obtained model in our study shows the intercept at *R*_*Y*_^2^ = 0.0487, *Q*_*Y*_^2^ = −0.355 (in Fig. S2 in ESI†), signifying the validity of the model. This shows that the developed model is non-random and robust, and is suitable for prediction of the larvicidal activity of compounds within the AD of the model.

### 3.5. Comparison with previously published models

We compared the currently developed model with previously developed models^7,20^ for larvicidal activity against *Aedes aegypti* in terms of quality measures (Table 2). However, due to the different compositions of the training and test sets in these studies, a critical comparison of the models is not possible. The advantage of the current model is that it has been developed by using simple 2D ETA descriptors which do not require conformation analysis or energy minimization prior to their calculation. Also, these descriptors have been calculated using freely available software (PaDel-Descriptor).^23^ The model we developed using a single class of descriptors (ETA) is comparable to or of better quality than those developed previously^7,20^ using computationally more expensive 3D descriptors.

**Table 2:** Comparison of current model with previously developed models

Models	Total no. of compounds used	No. of compounds in the training set	No. of compounds in the test set	Descriptor type	Number of descriptors used in the initial pool	*R* ^2^	No. of descriptors in the final model	*Q* ^2^ (LOO)	*Q*_*F*_1__^2^	*S* (train)	*S* (test)
Current study	61	41	20	2D	42	0.726	5 (3 LVs)	0.635	0.672	0.269	0.333
Saavedra *et al.*, 2018 (ref. 7)	62	52	10	2D + 3D	4885	0.690	5	0.600	—	0.28	0.39
Scotti *et al.*, 2014 (ref. 20)	55	41	14	3D	128	0.714	6	0.679	0.623	—	—

## 4. Conclusion

The present research used chemometric tools for investigating a set of 61 compounds of natural origin showing larvicidal activity against the zika vector *Aedes aegypti*. Based on the information obtained from the final PLS model (as also illustrated in the regression coefficient plot, variable importance plot, loading plot and score plot, in Fig. 1, 2, 8 and 9), we can conclude that: (i) the presence of hydrogen bond donor groups like –OH, –NH_2_, –SH *etc.* will attenuate the larvicidal activity against the zika vector; (ii) heteroatoms and multiple bonds are essential to increase the activity; (iii) the presence of electronegative hydrogen bond acceptor atoms helps to increase the larvicidal activity; (iv) a higher polar surface area is detrimental to the activity. The QSAR model developed here with simple and interpretable descriptors highlights the structural requirements and molecular properties needed to be present in the compounds for them to show acceptable larvicidal properties. The topological descriptors used also do not require the application of time-consuming computational procedures like conformational analysis or energy minimization; thus the developed model may be suitable for the quick screening of database compounds. The developed model further helps in the prediction of the activity of new analogues even before their synthesis and/or evaluation.

## Conflicts of interest

There are no conflicts to declare.

## Supplementary Material

RA-008-C7RA13159C-s001
