# Supplementary material for: Chemometric modeling of larvicidal activity of plant derived compounds against zika virus vector Aedes aegypti: application of ETA indices
Source: RSC Adv. 2018 Jan 25;8(9):4662–70. doi: 10.1039/c7ra13159c (PMC9077860; doi:10.1039/c7ra13159c)

Electronic Supplementary Material (ESI) for RSC Advances.
This journal is © The Royal Society of Chemistry 2018

Chemometric modeling of larvicidal activity of plant derived compounds against zika virus vector *Aedes aegypti*: Application of ETA indices

Priyanka De, Rahul B Aher and Kunal Roy*

Drug Theoretics and Cheminformatics Laboratory,

Department of Pharmaceutical Technology,

Jadavpur University, Kolkata 700 032, India,

Email: kunalroy_in@yahoo.com ; kunal.roy@jadavpuruniversity.in

Phone: +91 98315 94140; Fax: +91-33-2837-1078;

URL: http://sites.google.com/site/kunalroyindia/

SUPPLEMENTARY MATERIALS

Table S1. Molecular structures of the compounds used with their larvicidal activity data against *Aedes aegypti.*

Compound no.	Compound name	Structure	LC50 in ppm	pLC50 LC(mol/L)	Reference
1	(-)-Camphene	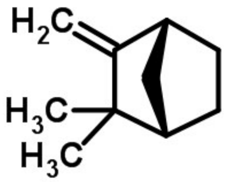	220	-	Santos, 2010
2	(±)-camphor	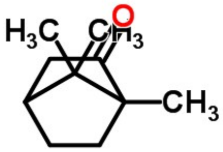	657	-	Santos,2010
3	1,4-cineole	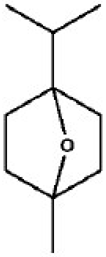	751	2.3	Santos,2010<br>Scotti,2014
4	1,8-cineole	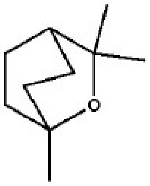	1419	2.04	Santos,2010<br>Scotti,2014
5	carvacrol	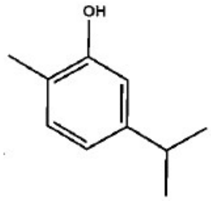	69	3.47	Santos,2010<br>Scotti,2014

6	carvacryl acetate	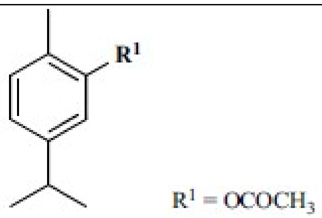	-	3.32	Scotti,2014
7	carvacryl chloroacetate	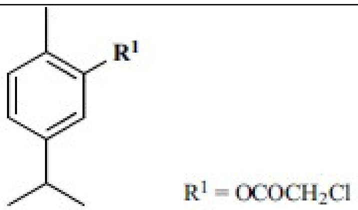	-	3.64	Scotti,2014
8	carvacryl trichloroacetate	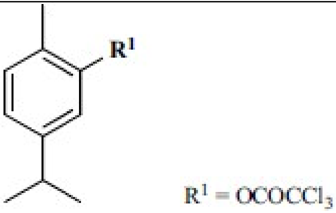	-	3.59	Scotti,2014
9	carvacryl propionate	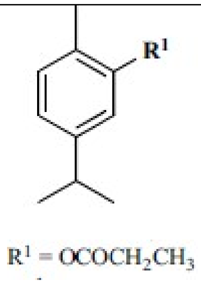	-	3.49	Scotti,2014
10	carvacryl benzoate	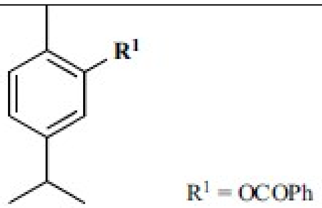	-	3.66	Scotti,2014
11	2-Hydroxy-3-methyl-6-(1-methylethyl)-benzaldehyde	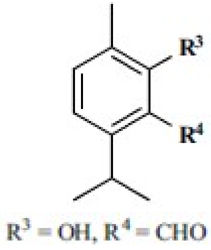	-	3.43	Scotti,2014

12	carvacrylglycolic acid	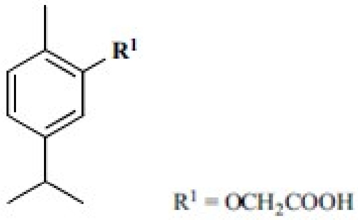	-	3.09	Scotti,2014
13	thymyl acetate	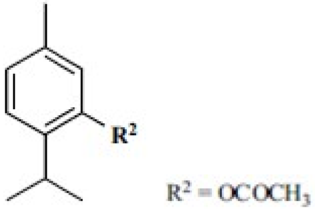	-	3.32	Scotti,2014
14	thymyl chloroacetate	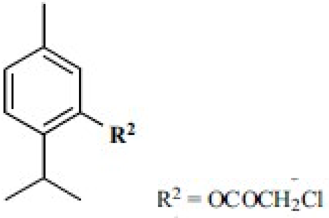	-	3.66	Scotti,2014
15	thymyl trichloroacetate	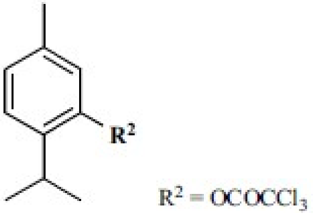	-	3.85	Scotti,2014
16	thymyl propionate	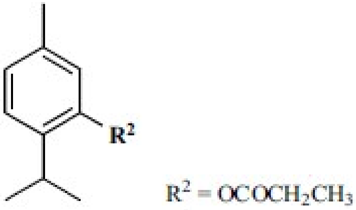	-	3.49	Scotti,2014
17	thymyl benzoate	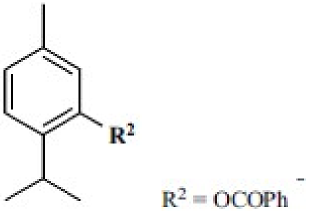	-	3.46	Scotti,2014

18	thymyl ethyl ether	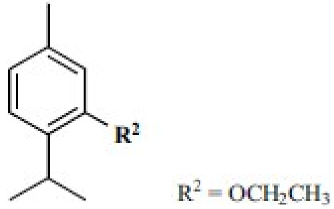	-	3.16	Scotti,2014
19	2-hydroxy-6-methyl-3-(1-methylethyl)-benzaldehyde	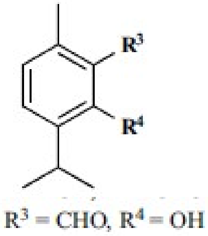	-	3.72	Scotti,2014
20	thymoxyacetic acid	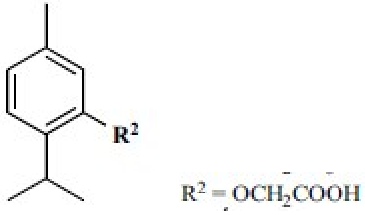	-	2.65	Scotti,2014
21	5-norbornene-2-ol	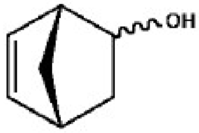	759	2.16	Santos,2010<br>Scotti,2014
22	5-norbornene-2,2-dimethanol	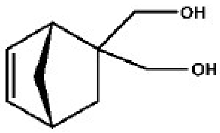	785	2.29	Santos,2010<br>Scotti,2014
23	5-norbornene-2-endo-3-endodimethanol	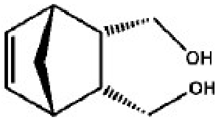	1407	2.04	Santos,2010<br>Scotti,2014
24	5-norbornene-2-exo-3-exo-dimethanol	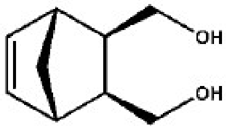	717	2.33	Santos,2010<br>Scotti,2014

25	eugenyl acetate	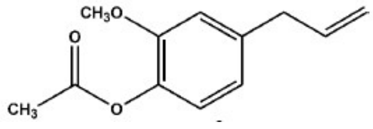	113.3	3.28	Barbosa,2010<br>Scotti,2014
26	2-[2-methoxy-4-(2-propen-1-yl)phenoxy] acetic acid	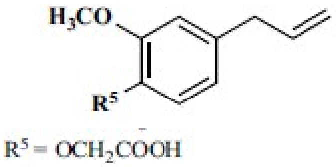	295.9	3.04	Barbosa,2010<br>Scotti,2014
27	borneol	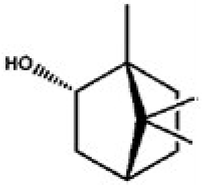	610	2.40	Santos 2010
28	Catechol	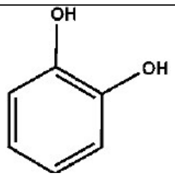	243	2.66	Santos 2010
29	1-ethoxy-2-methoxy-4-(2-propen-1-yl)-benzene	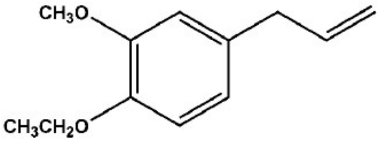	67.2	3.40	Barbosa 2012
30	Eugenol	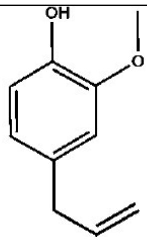	88, 93.3	3.35	Santos 2010, Barbosa 2012, Scotti 2014
31	Phenol	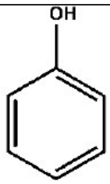	194	2.69	Santos 2010
32	gamma-terpinene	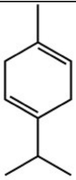	56	3.39	Santos 2011

33	guaiacol	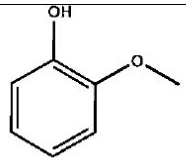	177	2.84	Santos 2010
34	1-benzoate-2-methoxy-4-(3-hydroxypropyl)-phenol	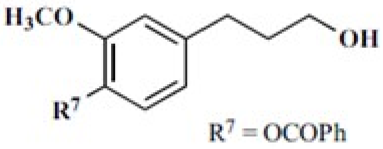	-	3.28	Scotti
35	4-hydroxy-3-methoxy-benzenepropanol	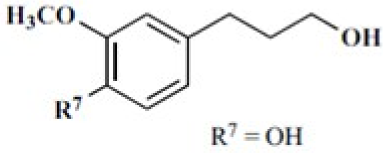	-	2.05	Scotti
36	isoborneol	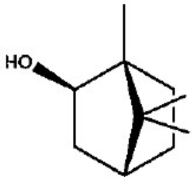	598	2.41	Santos 2010, Scotti
37	(-)-isopulegol	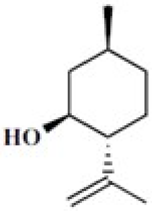	297	2.71	Santos 2011, Scotti
38	Thymol	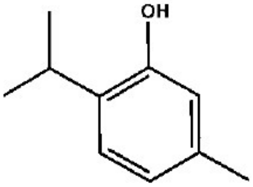	81	2.59	Santos 2010, Scotti
39	menthone	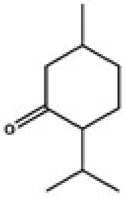	508	2.48	Santos 2011, Scotti

40	nonan-2-one (40),	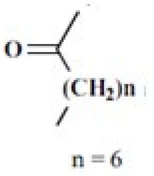	-	2.85	Scotti
41	undecan-2-one (41),	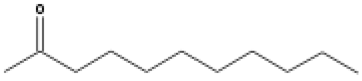	-	3.51	Scotti
42	1,2-dimethoxy-4-(2-propen-1-yl)-benzene	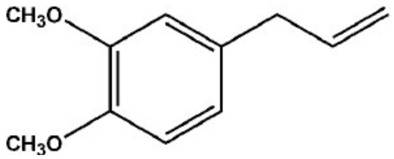	107.3	3.24	Barbosa 2012, Scotti
43	neoisopulegol	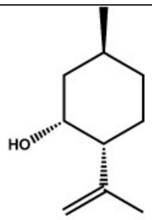	554	2.44	Santos 2011, Scotti
44	1,2-carvone oxide	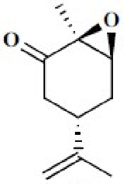 44	219	2.88	Santos 2011, Scotti
45	Limonene oxide, mixture of cis and trans	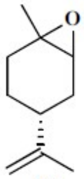 45	517	2.47	Santos 2011, Scotti
46	*p*-cymene	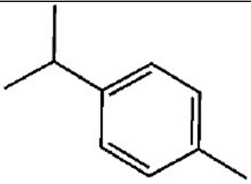	51	3.42	Santos 2010, Scotti
47	eugenyl propionate	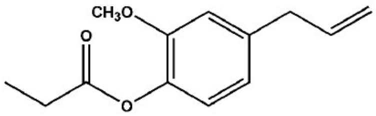	97.2	3.55	Barbosa 2012, Scotti

48	R-carvone	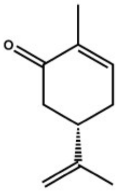	152	3.00	Santos 2011, Scotti
49	resorcinol	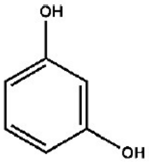	577	2.28	Santos 2010, Scotti
50	R-limonene	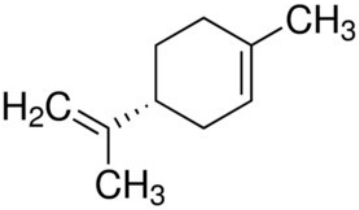	27	3.70	Santos 2011, Scotti
51	R/S-carvone	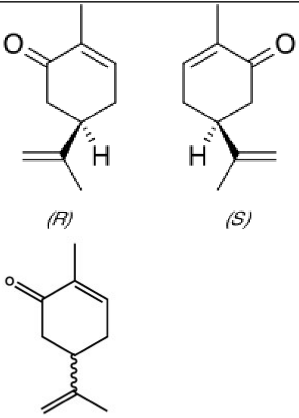	118	3.11	Santos 2011, Scotti
52	Salicylaldehyde	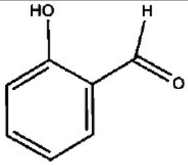	136	2.95	Santos 2010, Scotti
53	S-Carvone	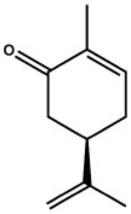	124	3.08	Santos 2011, Scotti

54	S-limonene	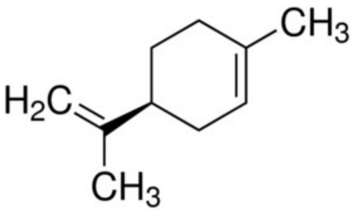	30	3.64	Santos 2011, Scotti
55	vanillin	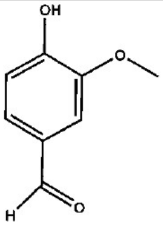	513	2.47	Santos 2010, Scotti
56	(1R,2R,5R)-2-Methyl-5-(1-methylethenyl)-3-oxo-cyclohexanecarbonitrile	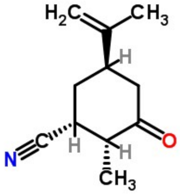	412	-	Santos,2011
57	1-Benzoate-2-methoxy-4-(2-propen-1-yl)-Phenol	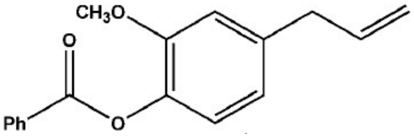	706.8	-	Barbosa 2012
58	1-{[(1,1-Dimethylethyl)dimethylsilyl]oxy}-2-methoxy-4-(2-propen-1-yl)-benzene	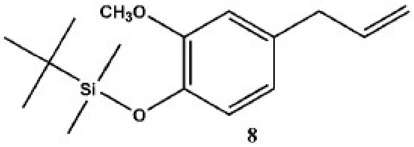 8	278.9	-	Barbosa 2012
59	3-Carene	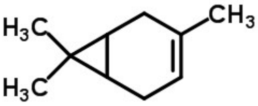	150	-	Santos,2011

60	Menthol	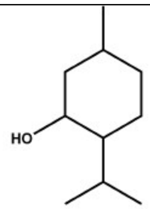	404	-	Santos,2011
61	(+)-Camphene	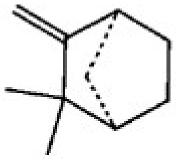	406	-	Santos,2010

Table S2. The values of descriptors appearing in Eq. (1) and computed larvicidal activity values for both training and test set compounds

Sl. No.	ETA_EtaP_F	ETA_dEpsilon_D	ETA_dAlpha_B	ETA_BetaP_s	ETA_dEpsilon_C	pLC50(obs)	pLC50(pred)
Training set							
2	0.221	0	0.015	0.568	-0.049	2.36	2.67
3	0.107	0	0.015	0.591	-0.036	2.31	2.36
5	0.573	0.039	0.015	0.523	-0.033	3.34	3.14
7	0.662	0	0.008	0.567	-0.095	3.64	3.42
8	0.67	0	0	0.588	-0.155	3.59	3.53
10	0.848	0	0.018	0.566	-0.048	3.66	3.42
11	0.704	0.037	0.026	0.538	-0.071	3.43	3.16
13	0.666	0	0.024	0.554	-0.065	3.32	3.22
14	0.665	0	0.008	0.567	-0.095	3.66	3.42
16	0.647	0	0.022	0.55	-0.06	3.49	3.24
17	0.852	0	0.018	0.566	-0.048	3.46	3.42
18	0.537	0	0.013	0.538	-0.028	3.16	3.23
20	0.696	0.035	0.033	0.567	-0.091	2.65	2.93
21	0.234	0.055	0.021	0.594	-0.053	2.16	2.16
23	0.297	0.083	0.03	0.591	-0.078	2.04	2.04
24	0.297	0.083	0.03	0.591	-0.078	2.33	2.04
25	0.808	0	0.033	0.583	-0.091	3.26	3.15
28	0.709	0.118	0.042	0.562	-0.103	2.66	2.43
29	0.699	0	0.024	0.571	-0.054	3.46	3.13
31	0.632	0.064	0.024	0.536	-0.054	2.69	2.93
32	0.235	0	0	0.5	0	3.39	3.20
33	0.7	0.057	0.037	0.583	-0.09	2.85	2.66
35	0.668	0.077	0.038	0.577	-0.094	2.05	2.54
36	0.101	0.039	0.015	0.568	-0.036	2.41	2.29
37	0.244	0.039	0.015	0.523	-0.033	2.72	2.73
39	0.219	0	0.015	0.523	-0.045	2.48	2.94
40	0.204	0	0.017	0.475	-0.045	2.85	3.19
42	0.731	0	0.026	0.577	-0.059	3.22	3.12
44	0.422	0	0.028	0.604	-0.085	2.88	2.60
45	0.235	0	0.015	0.591	-0.036	2.47	2.52
46	0.496	0	0	0.5	0	3.42	3.53
49	0.705	0.118	0.042	0.562	-0.103	2.28	2.43

50	0.265	0	0	0.5	0	3.7	3.24
51	0.479	0	0.015	0.523	-0.045	3.1	3.26
52	0.754	0.057	0.037	0.556	-0.111	2.95	2.94
53	0.479	0	0.015	0.523	-0.045	3.08	3.26
57	0.959	0	0.025	0.588	-0.068	2.58	3.37
58	0.663	0	0	0.592	-0.025	3	3.22
59	0.116	0	0	0.55	0	2.96	2.75
60	0.1	0.039	0.015	0.523	-0.033	2.59	2.56
61	0.161	0	0	0.55	0	2.53	2.80
Test set							
1	0.161	0	0	0.55	0	2.79	2.8
4	0.11	0	0.015	0.591	-0.036	2.04	2.36
6	0.644	0	0.022	0.55	-0.06	3.32	3.24
9	0.644	0	0.022	0.55	-0.06	3.49	3.24
12	0.693	0.035	0.033	0.567	-0.091	3.09	2.92
15	0.673	0	0	0.588	-0.155	3.85	3.54
19	0.703	0.037	0.026	0.538	-0.071	3.72	3.15
22	0.306	0.083	0.03	0.591	-0.078	2.29	2.05
26	0.831	0.035	0.042	0.594	-0.116	2.88	2.86
27	0.101	0.039	0.015	0.568	-0.036	2.4	2.29
30	0.737	0.041	0.028	0.562	-0.065	3.26	2.99
34	0.908	0.025	0.032	0.595	-0.087	3.28	3.07
38	0.575	0.039	0.015	0.523	-0.033	3.27	3.14
41	0.179	0	0.014	0.479	-0.037	3.51	3.16
43	0.244	0.039	0.015	0.523	-0.033	2.44	2.73
47	0.788	0	0.031	0.578	-0.084	3.36	3.17
48	0.479	0	0.015	0.523	-0.045	2.99	3.26
54	0.265	0	0	0.5	0	3.66	3.24
55	0.806	0.051	0.045	0.591	-0.135	2.47	2.76
56	0.542	0	0.021	0.538	-0.079	2.63	3.24

Figure S1: DModX value of training set and test set compounds at 99% confidence level for the developed PLS model. The thick horizontal line signifies the critical DModX value (0.00999898) at the 99% confidence level.

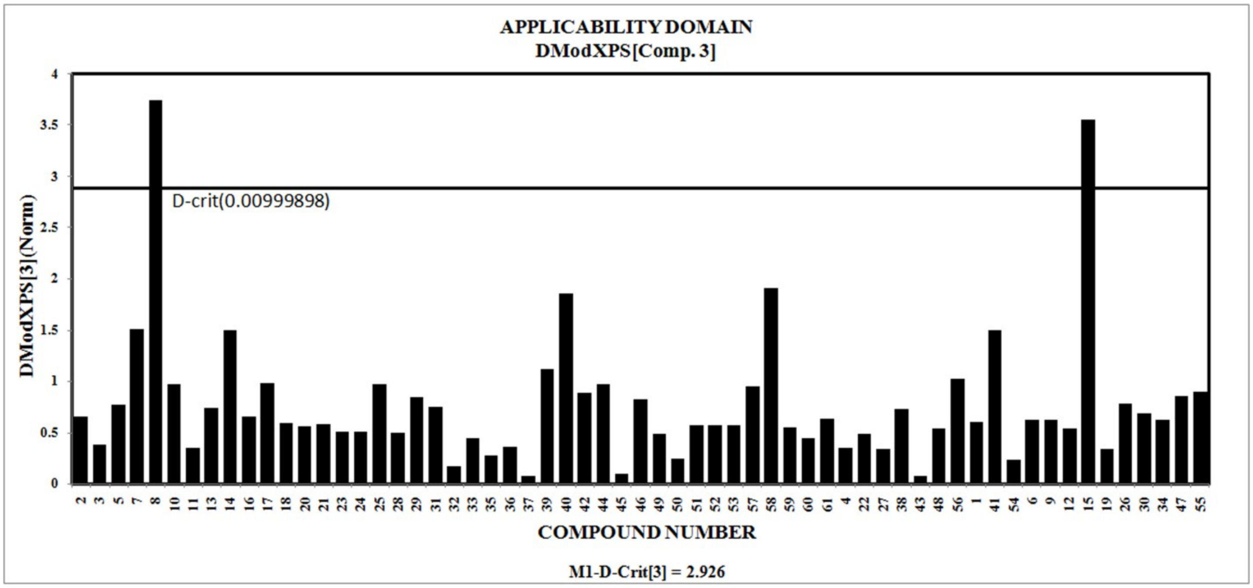

Figure S2: Randomization model for final PLS model

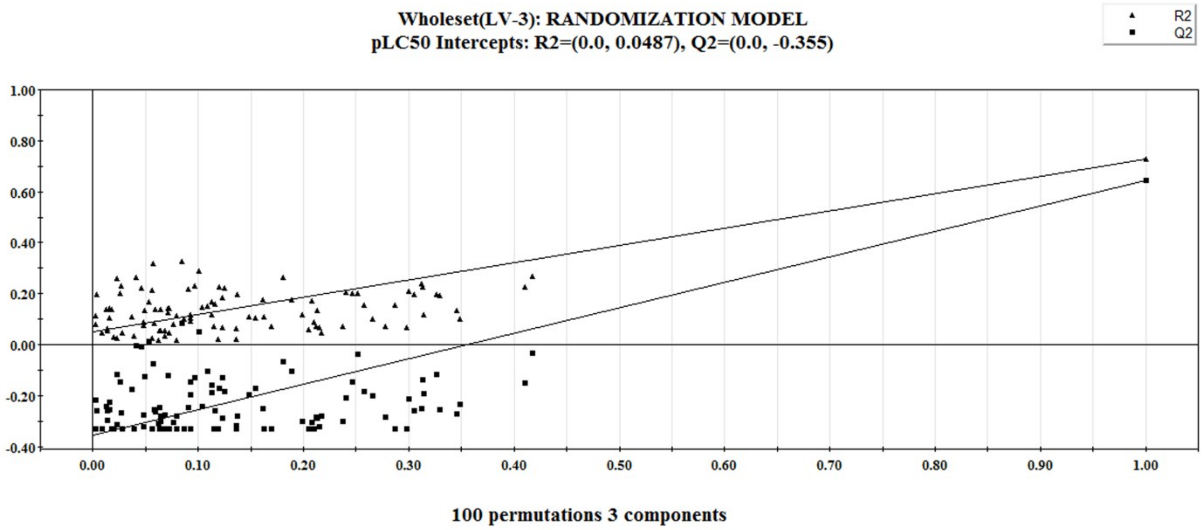

Supplement: RA-008-C7RA13159C-s001 [file RA-008-C7RA13159C-s001.pdf]
